# Diagnostic Accuracy of Acid-Base Status in Infants with Hypertrophic Pyloric Stenosis

**DOI:** 10.3390/children9121815

**Published:** 2022-11-24

**Authors:** Marko Bašković, Dorotea Sinjeri

**Affiliations:** 1 Department of Pediatric Surgery, Children’s Hospital Zagreb, Ulica Vjekoslava Klaića 16, 10000 Zagreb, Croatia; 2 Scientific Centre of Excellence for Reproductive and Regenerative Medicine, School of Medicine, University of Zagreb, Šalata 3, 10000 Zagreb, Croatia; 3 School of Medicine, University of Zagreb, Šalata 3, 10000 Zagreb, Croatia; 4 Department of Pediatric Surgery, University Hospital Centre Zagreb, Kišpatićeva Ulica 12, 10000 Zagreb, Croatia

**Keywords:** hypertrophic pyloric stenosis, acid-base status, electrolytes, ultrasound, diagnostic, vomit, newborn, infant, pediatric surgery

## Abstract

Background: Hypertrophic pyloric stenosis is a condition in newborns in which the hypertrophic pyloric muscle causes gastric obstructive symptoms of progressive vomiting leading to hypochloremic hypokalemic metabolic alkalosis. The main aim of the research was to assess whether, based on the acid-base status, we can distinguish newborns who vomit due to pylorostenosis, compared with newborns who vomit for other unspecific reasons. Methods: The electronic records of patients in the hospital information system treated under the diagnosis Q40.0 (Congenital hypertrophic pyloric stenosis) (*n* = 69/included in the study = 53) in the period from 1 January 2014 to 1 January 2022 were reviewed retrospectively. For the purposes of the control group, the electronic records of patients treated in the emergency department with a diagnosis of R11.0 (Nausea and vomiting) (*n* = 53) without an established cause were randomly reviewed. In addition to the main aim, other research outcomes were to determine differences between groups in the following variables: duration of symptoms, family history, birth (preterm, term, post-term), birth weight, weight during examination, difference between birth weight and weight during an examination, type of vomiting, the thickness of the muscle wall and its length, and to calculate whether there is a correlation between the thickness and length of the pylorus muscle and the duration of vomiting in relation to variables from acid-base status. Results: In relation to the variables of interest between the groups, statistically significant differences were observed in the duration of symptoms (Mdn 4 vs. 2 days, *p* = 0.002), weight at examination (Mean 3880 vs. 4439 g, *p* = 0.001), difference in weight between birth and examination (Mean 374 vs. 1010 g, *p* < 0.0001), and type of vomiting (explosive 45 vs. 22, *p* = 0.023). In the acid-base status between the groups, a statistically significant difference was recorded for pH (Mdn 7.457 vs. 7.422, *p* < 0.0001), bicarbonate (Mdn 25 vs. 23 mmol/L, *p* = 0.000), total carbon dioxide (Mdn 25 vs. 24 mmol/L, *p* = 0.011), base excess (Mdn 0.8 vs. −1.3 mmol/L, *p* = 0.000), potassium (Mdn 5 vs. 5.3 mmol/L, *p* = 0.006), ionized calcium (Mdn 1.28 vs. 1.31 mmol/L, *p* = 0.011), and glucose (Mdn 4.5 vs. 4.9 mmol/L, *p* = 0.007). Regardless of the group, the correlations between the duration of vomiting (r = 0.316, *p* = 0.021 vs. r = 0.148, *p* = 0.290) and the thickness (r = 0.190, *p* = 0.172) and length (r = 0.142, *p* = 0.311) of the pylorus muscle in relation to pH did not exist or were weak. Conclusions: In a world where radiological methods are not equally available everywhere, with promising acid-base indicators, prospective multicenter studies and meta-analyses must be pursued in the future in order not to miss the possible much greater diagnostic potential of acid-base status.

## 1. Introduction

Regulation of the balance of hydrogen ions (H^+^) is extremely important because the activity of almost all enzyme systems depends on the concentration of hydrogen ions. If acid-base balance disorders are primarily caused by a change in the extracellular concentration of hydrogen carbonate (HCO_3_^−^), we are talking about metabolic acid-base disorders. Vomiting only stomach contents, without contents from the lower parts of the digestive system, leads to the loss of hydrochloric acid (HCl) secreted by the gastric mucosa. The result is the loss of acid from the extracellular fluid and the development of metabolic alkalosis. This type of alkalosis is primarily specific to newborns with hypertrophic pyloric stenosis. Treatment of metabolic alkalosis requires suppression of the primary cause of vomiting itself and compensation of sodium, potassium, and chloride deficits, which enables the optimal functioning of the renal corrective mechanisms. Just by giving a sodium chloride (NaCl) solution in which sodium and chloride are in an equivalent ratio, we introduce a relative excess of chloride into the body, which pushes hydrogen carbonate out of the anion column of the ionogram and corrects alkalosis [1,2,3].

Hypertrophic pyloric stenosis is one of the most common surgical conditions in children of the earliest age, which typically occurs between the 2nd and 12th week of life [4], but cases have also been recorded outside of this period [5]. It is more common in male children in a ratio of 4–6:1 and the median is around the 40th day of life [6]. The incidence of the disease is 2–4 per 1000 live births with a higher frequency in temperate countries than in the equatorial region, which indicates a connection between environmental factors and the development of the disease [7]. Although the cause of pyloric stenosis is still a matter of speculation, the exact cause is still unknown, but it is most likely multifactorial [8,9,10,11,12]. The generally accepted method, as the gold standard in diagnosis, is ultrasound with a sensitivity of 99.5% and a specificity of 100%. Ultrasound criteria for diagnosis are thickening of the pyloric muscle > 3 mm and length of the pyloric canal > 15 mm [13,14]. Misdiagnosis is possible in children who have pylorospasm, and that is why it is advisable to do an X-ray contrast examination of the stomach and duodenum if the clinical picture is unclear. Rehydration and correction of electrolyte abnormalities are required before surgery. At the time of arrival at the hospital, most patients require 20 mL/kg of saline as a bolus, once or repeatedly, and then continuously intravenously at 4–6 mL/kg/h until the electrolyte imbalance is corrected. Response to therapy is achieved within 12–48 h, after which surgery is recommended. Uncorrected alkalosis can adversely affect the respiratory function of neonates in the perioperative period, and it has been proven that the degree of alkalosis at admission strongly correlates with the number of episodes of postoperative vomiting and the length of recovery [15,16]. The surgical approach includes pyloromyotomy, which may be open or laparoscopic. A regular incision extends from the prepyloric vein at the junction with the duodenum to the circular muscle fibers of the distal part of the antrum, proximal to the pylorus [4,6,17,18]. Laparoscopy is an efficient and reliable alternative with an increased risk of incomplete pyloromyotomy and perforation of the mucosa, but the duration of hospitalization is shorter and recovery is faster, so today it is increasingly replacing the classical approach [19,20,21,22].

This single-center, retrospective, cross-sectional study aimed to evaluate acid-base status values in order to distinguish neonates who vomited due to hypertrophic pyloric stenosis versus neonates who vomited without a proven cause.

## 2. Materials and Methods

### 2.1. Patients

For the purpose of the research, electronic records of patients in the hospital information system (IN2 BIS^®^) were reviewed retrospectively. The search included all patients who were treated at the Children’s Hospital Zagreb with a diagnosis of Q40.0 (Congenital hypertrophic pyloric stenosis—according to the International Classification of Diseases 10th Revision) in the period from 1 January 2014 to 1 January 2022. The inclusion criterion was all patients who underwent surgery in whom the diagnosis was confirmed during the surgery, and the exclusion criterion was the lack of data for analysis. All operations were performed using an open surgical technique (after upper mini-midline laparotomy, the pylorus is identified and delivered outside the abdomen. The prepyloric vein of Mayo is identified, which marks the pyloroduodenal junction, and the proximal end is recognized by the palpable end of the hypertrophic muscle reaching the antrum of the stomach. A longitudinal seromuscular incision is made that extends along the entire length of the hypertrophic muscle. This is followed by the spreading of the pyloric muscle until the protrusion of the intact mucosa is verified. After adequate hemostasis, the abdominal wall is closed in layers). For the purposes of the control group, the electronic records of patients treated in the emergency department of the Children’s Hospital Zagreb with a diagnosis of R11.0 without an established cause (Nausea and vomiting—according to the International Classification of Diseases 10th Revision) were also randomly reviewed in such a way that they were comparable in terms of gender and ages with group Q40.0. In this group, patients older than three months and patients who lacked the necessary data for analysis were excluded.

### 2.2. Study Design

Demographic and anamnestic data, including sex; age; family history (of hypertrophic pyloric stenosis); duration of symptoms; birth before, at, or after term; birth weight, weight at the examination, and their difference; and type of vomiting were recorded for each patient. In the Q40.0 group, the thickness of the pyloric muscle and its length were also recorded. Regarding the acid-base status, the values of the following variables were recorded: pH, pCO_2_, pO_2_, HCO_3_, total CO_2_, base excess, sO_2_, potassium, sodium, chloride, ionized calcium, glucose, lactate, total bilirubin, oxyhemoglobin, carboxyhemoglobin, methemoglobin, and deoxyhemoglobin.

### 2.3. Diagnostic Procedures

The acid-base status of the patients was measured from capillary blood after a sample was taken professionally by a laboratory technician. In all patients, capillary blood was taken within 3 h of the examination. Blood was analyzed on RAPIDPoint^®^ 500 Systems (Siemens Healthcare GmbH, Erlangen, Germany). Abdominal ultrasound with a targeted examination of the pyloric region was performed by one of the five radiologists who participated in the diagnosis of this condition in the past 8 years. If the first ultrasound finding was not clear due to artifacts (e.g., stomach filled with gas, stomach filled with milk, markedly distended stomach, etc.), it was repeated (sometimes twice) within 24 h, until the final thickness and length of the pyloric muscle were determined with great certainty. All ultrasound examinations were performed on a Logiq E9^®^ device (GE Healthcare Systems, Chicago, IL, USA).

### 2.4. Outcome Measures

The main outcome of the study was to determine whether the values from the acid-base status, regardless of the ultrasound performed, can suggest that a patient is a newborn with hypertrophic pyloric stenosis rather than a newborn who is vomiting for some other unspecified reason. The secondary outcomes of the study were to determine at what age parents brought newborns with symptoms of hypertrophic pyloric stenosis; whether there was an association with family history; whether the newborns were born before or at term; duration of symptoms; birth weight and weight at clinical examination, and the difference in weight in the same period; whether explosive vomiting dominated in relation to regurgitation; the thickness of the muscle wall and its length; and, in addition, to calculate whether there is a correlation between the thickness of the pylorus muscle and the duration of vomiting in relation to variables from acid-base status.

### 2.5. Statistical Analysis

Descriptive statistics were used to characterize the patient cohort. Collected measurements were analyzed for normal distribution using the Shapiro–Wilk test. Categorical variables were expressed in absolute numbers and percentages. Fisher’s exact test was used to assess differences in the distribution of categorical data. Continuous variables were expressed as mean with standard deviation (SD) and median (Mdn) with interquartile range (IQR) and were analyzed using the Student’s *t*-test or Mann–Whitney U test as appropriate. In order to calculate the correlation, Pearson’s or Spearman’s correlation coefficient was calculated, depending on the distribution normality, and the results were interpreted according to the following intervals: 0–±0.25 ↔ no correlation, ±0.26–±0.50 ↔ weak correlation, ±0.51–±0.75 ↔ good correlation, ±0.76–±1 ↔ excellent correlation. A multiple regression analysis was also performed for the data of interest. The obtained data were analyzed using the Microsoft Excel^®^ software program (XLSTAT^®^) for Windows, version 2020.5.1 (Microsoft Corporation, Redmond, WA, USA). A significance level of 0.05 was used.

## 3. Results

During the selected period, a total of 69 newborns with a diagnosis of hypertrophic pyloric stenosis (Q40.0) were surgically treated at the Children’s Hospital Zagreb. Complete documentation for all variables of interest was missing in 16 patients, and thus a total of 53 patients were included in the study. For the control group, 53 patients (R11.0) comparable in terms of gender (*p* = 1.000) and age (*p* = 0.674) were randomly selected.

### 3.1. Pylorostenosis Group (Q40.0)

In the mentioned period, a total of 41 (77.36%) male and 12 (22.64%) female children were surgically treated. The median age at which children presented to the emergency department with symptoms of hypertrophic pyloric stenosis was 31 (IQR 24, 44) days. Only one patient (1.89%) had information in the family history stating that one of the parents also had pyloric stenosis. Five children (9.43%) were born before term, whereas forty-eight (90.57%) were born at term. The median duration of symptoms was 4 (IQR 2, 7) days. The mean birth weight was 3506 (±558) g, and the mean weight at examination was 3880 (±601) g. The mean difference in weight from birth to examination was 374 (±568) g. Forty-five (84.91%) children presented with explosive vomiting, and eight (15.09%) with regurgitation. The mean thickness of the pyloric muscle was 4.857 (±0.879) mm, whereas the median was 4.9 (IQR 4.3, 5.4) mm. The mean muscle length was 17.317 (±3.809) mm, whereas the median was 17 (IQR 15, 19).

### 3.2. Control Group (R11.0)

In the randomly selected patients who made up this group, there were also 41 (77.36%) male and 12 (22.64%) female children. The median age at which children presented to the emergency department with symptoms of vomiting without an identified cause was 35 (IQR 21, 50) days. None of the patients had information about hypertrophic pyloric stenosis in their family history. Six children (11.32%) were born before term, whereas forty-seven (88.68%) were born at term. The median duration of symptoms was 2 (IQR 1, 5) days. The mean birth weight was 3428 (±525) g, whereas the mean weight at examination was 4439 (±1025) g. The mean difference in weight from birth to examination was 1010 (±858) g. Thirty-one (58.49%) children presented with regurgitation, whereas twenty-two (41.51%) presented with explosive vomiting.

### 3.3. Differences between Groups

According to the previously mentioned variables, statistically significant differences were observed in family history, duration of symptoms, weight at examination, the difference in weight between birth and examination, and type of vomiting (Table 1).

### 3.4. Differences in Acid-Base Status between Groups

In relation to the observed groups, a statistically significant difference was recorded for the following variables from the acid-base status: pH, bicarbonate (HCO_3_), total carbon dioxide (CO_2_), base excess (BE), potassium (K^+^), ionized calcium (Ca^2+^), and glucose (Table 2).

### 3.5. Correlation between Thickness and Length of Pyloric Muscle and Values of Acid-Base Status

**Figure 1:**
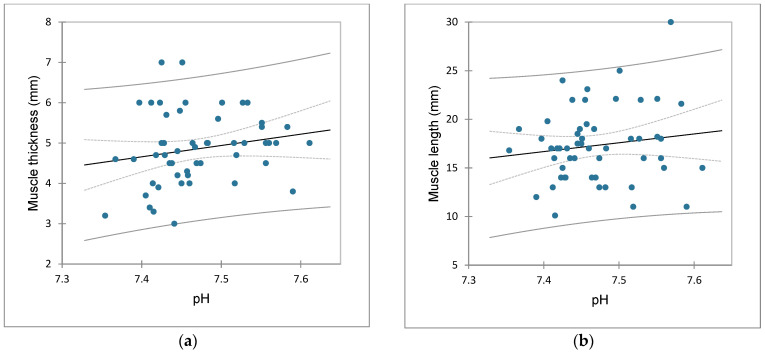
Scatterplots: (**a**) correlation of muscle thickness and pH; (**b**) correlation of muscle length and pH.

The correlation values of interest were as follows: muscle thickness ↔ pH (r = 0.190, *p* = 0.172) (Figure 1a), muscle length ↔ pH (r = 0.142, *p* = 0.311) (Figure 1b), muscle thickness ↔ pCO_2_ (r = 0.124, *p* = 0.375), muscle thickness ↔ HCO_3_ (r = 0.322, *p* = 0.019), muscle thickness ↔ total CO_2_ (r = 0.280, *p* = 0.043), muscle thickness ↔ BE (r = 0.308, *p* = 0.025), muscle thickness ↔ potassium (r = −0.350, *p* = 0.010), muscle thickness ↔ sodium (r = −0.303, *p* = 0.027), muscle thickness ↔ chloride (r = −0.247, *p* = 0.075), muscle thickness ↔ ionized calcium (r = −0.103, *p* = 0.462).

In relation to the previous parameters, multiple regression analysis determined that 33% of the dependent variable (muscle thickness) was explained by nine explanatory variables. Among the explanatory variables, based on the sum of squares type III, sodium (t = −2.971, *p* = 0.005) and potassium (t = −2.339, *p* = 0.024) proved to be the most influential variables.

### 3.6. Correlation between Duration of Vomiting and Acid-Base Status

**Figure 2:**
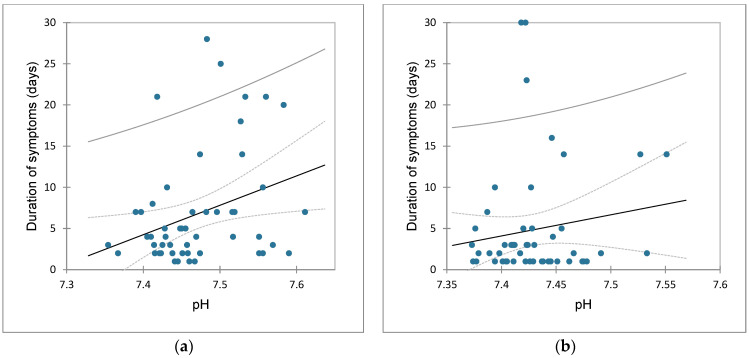
Scatterplots: (**a**) correlation of duration of vomiting and pH in the pylorostenosis group; (**b**) correlation of duration of vomiting and pH in the control group.

The correlations of values from acid-base status and duration of vomiting in the pylorostenosis group were as follows: ↔ pH (r = 0.316, *p* = 0.021) (Figure 2a), ↔ pCO_2_ (r = 0.216, *p* = 0.120), ↔ HCO_3_ (r = 0.532, *p* < 0.0001), ↔ total CO_2_ (r = 0.493, *p* = 0.000), ↔ BE (r = 0.512, *p* < 0.0001), ↔ potassium (r = −0.505, *p* = 0.000), ↔ sodium (r = −0.273, *p* = 0.048), ↔ chloride (r = −0.418, *p* = 0.002), ↔ ionized calcium (r = −0.212, *p* = 0.128). In relation to the previous parameters, through multiple regression analysis, it was determined that 47% of the dependent variable (the duration of vomiting) was explained by nine explanatory variables. Among the explanatory variables, based on the sum of squares of type III, sodium (t = −2.440, *p* = 0.019) and potassium (t = −2.767, *p* = 0.008) proved to be the most influential variables.

The correlations of values from acid-base status and duration of vomiting in the control group were as follows: ↔ pH (r = 0.148, *p* = 0.290) (Figure 2b), pCO_2_ (r = 0.163, *p* = 0.245), ↔ HCO_3_ (r = 0.253, *p* = 0.067), ↔ total CO_2_ (r = 0.280, *p* = 0.042), ↔ BE (r = 0.288, *p* = 0.037), ↔ potassium (r = 0.036, *p* = 0.801), ↔ sodium (r = 0.025, *p* = 0.858), ↔ chloride (r = −0.138, *p* = 0.325), ↔ ionized calcium (r = 0.184, *p* = 0.188). In relation to the previous parameters, through multiple regression analysis, it was determined that 25% of the dependent variable (the duration of vomiting) was explained by nine explanatory variables. Among the explanatory variables, based on the sum of squares of type III, ionized calcium (t = 2.143, *p* = 0.038) proved to be the most influential variable.

### 3.7. Influence of Duration of Vomiting Longer and Shorter than Three Days on Acid-Base Status

The duration of symptoms longer than three days compared with the duration of symptoms shorter than three days had a statistically significant effect in both groups for an increase in bicarbonate, base excess, and total CO_2_, whereas a statistically significant decrease was recorded for potassium in the pylorostenosis group (Table 3 and Table 4).

## 4. Discussion

With this study, we determined certain variables that differ significantly between groups, which allows some conclusions to be drawn. Statistically significant differences were observed in family history, duration of symptoms, weight at examination, the difference in weight between birth and examination, and type of vomiting. In the acid-base status between the groups, a statistically significant difference was recorded for pH, bicarbonate, total carbon dioxide, base excess, potassium, ionized calcium, and glucose. Regardless of the group, the correlations between the duration of vomiting and the thickness and length of the pylorus muscle in relation to pH did not exist or were weak.

When looking exclusively at patients with pylorostenosis, there are similarities, but also differences, between the studies published so far. As for the median duration of symptoms, we find medians of 5, 6, and even 14 days [23,24,25]. In all studies, the male gender dominated, so we find dominance in ratios of 5:1, 6:1, and 7:1 [25,26,27,28]. A positive family history was recorded in 9.4% of cases, and the median age when patients presented to the doctor was 4, 5, and 6 weeks, respectively [25,26,28,29]. Prematurity was recorded in 6.4% of cases, and greater weight loss was also characteristic of the pylorostenosis group [26,30].

In a study by Al-Jazaeri et al. [24], patients with hypertrophic pyloric stenosis presented with the following values: mean chloride level 93.9 (±8.8) mEq/L, pH 7.5 (±0.9), and bicarbonate 27.8 (±6.4). The aim of these authors was to examine whether the duration of symptoms and the use of different techniques correlate with acid-base status, time of postoperative feeding, and duration of hospitalization. Ultimately, they did not observe a significant correlation. A study from Australia found that vomiting lasting longer than one week and a dehydration estimate greater than 5% will almost always result in a greater biochemical abnormality. The same study found that the average pH value was significantly higher in female newborns (*p* < 0.05), who were older with longer duration of symptoms at the time of presentation to the health facility. All parameters except urea were associated with the duration of symptoms. As the duration of symptoms increased, sodium, potassium, and chloride levels tended to decrease, whereas base excess, bicarbonate, and carbon dioxide tended to increase [26]. A study by Goh et al. [27] aimed to examine the correlation between chloride concentration in plasma and the degree of alkalemia and to determine whether chloride concentration can be used as a reliable parameter in the assessment and correction of alkalemia. They found that plasma chloride is a reliable parameter in the assessment and correction of alkalemia, provided that the target concentration is at least 106 mmol/L. They also determined that the average duration of symptoms in the normacidemic group was 8 days, whereas in the alkalemic group it was 13 days (*p* > 0.05). An interesting indicator was that in the normacidemic group no one had hypokalemia, whereas in the alkalemic group 15% of patients were hypokalemic (<3.5 mmol/L). For the above reason, the authors concluded that the use of sodium chloride is the mainstay of therapy, but in more severe cases, potassium supplementation is also needed. Two years after the previous study, Shanbhogue et al. [25], in addition to serum chloride concentration, also evaluated serum sodium, potassium, and bicarbonate concentrations in predicting acid-base status. Through multiple regression analysis, using bicarbonate as the dependent variable, they found that serum chloride concentration was the strongest independent predictor of bicarbonate level, whereas other variables did not significantly improve the correlation. They found that a serum chloride level of 106 mmol/l had a sensitivity of 71% and a specificity of 69% for identifying non-alkalemia versus alkalemia during admission, i.e., during the correction of fluid and electrolyte abnormalities. Although in our study a significant proportion of patients with hypertrophic pyloric stenosis did not have metabolic alkalosis (43.4%), according to the study by Tutay et al. [28], most of their patients with hypertrophic pyloric stenosis did not show alkalosis or electrolyte disturbances. They also determined that the duration of vomiting is not a significant predictor, but they confirmed that age at the time of diagnosis is associated with alkalosis. The proportion of cases with normal carbon dioxide was 62%, and the proportions with low and high carbon dioxide were 20% and 18%, respectively. The proportion with normal serum potassium was 57%, and the proportions with low and high serum potassium were 8% and 35%, respectively. The share with normal chloride was 69%, and the proportions with low and high chloride were 25% and 6%, respectively. Although there is no evidence in the currently available literature about the effectiveness of different fluid replacement regimens in patients with hypertrophic pyloric stenosis [13,31], researchers from the United Kingdom [29] found that there is a significant correlation (*p* < 0.05) between the degree of initial biochemical disturbance and the rate at which correction occurs if we stick to a certain protocol. The markers with the highest correlation and significance were chloride, urea, and base excess (*p* < 0.01).

The only study similar to ours was undertaken by Oakley et al. [30] more than 20 years ago. They compared the clinical and biochemical parameters of 100 patients with hypertrophic pyloric stenosis and 84 patients of a similar age who came to the emergency department with vomiting. Clinical correlates consisted of age, duration of vomiting, weight loss, gestation, and family history. Biochemical correlates were pH, bicarbonate, base excess, chloride, sodium, and potassium. Independent variables with significance were pH, base excess, chloride, bicarbonate, potassium, weight loss (*p* < 0.0001), and sex (*p* = 0.006). When the mentioned variables were put into a logistic regression with pyloric stenosis as the dominant variable, the variables of significance were pH (*p* = 0.0001), base excess (*p* = 0.0001), and chloride (*p* = 0.009). Based on these variables, a model was created to predict pyloric stenosis (pH > 7.45, chloride < 98, base excess > +3) with a specificity of 99%, a sensitivity of 40%, a positive predictive value of 88%, and a negative predictive value of 78% if all three variables were present. If two variables were present, the specificity was 98%, and the sensitivity was 65%, whereas with one variable, the specificity was 84%, and the sensitivity was 83%. There is no doubt that the sensitivity and specificity of ultrasound as the gold standard in the diagnosis of hypertrophic pyloric stenosis exceeds these values [32,33]. Except for sodium, all biochemical variables showed a significant difference between the groups. They also considered the effect of the duration of vomiting on biochemical parameters, where they found that the duration of symptoms caused a significant difference in pH, bicarbonate, base excess, and to a lesser extent in sodium. In the control group, there was no significant difference in any biochemical variable. In relation to the above results, in our study, observing the impact of vomiting longer and shorter than three days, we observed a statistically significant increase in bicarbonate, base excess, and total CO_2_ in both groups and a statistically significant decrease in potassium in the pylorostenosis group.

In the developed world, the acid-base status in a child with hypertrophic pyloric stenosis has started to be understood less and less as a possible diagnostic tool for pyloric stenosis itself, but primarily serves as an indicator of biochemical variables that should be corrected if there is a deviation, all in order to avoid depression of the respiratory center and apnea during and after the operation itself, especially if the child was hyperventilated during anesthesia [34,35]. Radiological methods, primarily ultrasound, have become the gold standard in the diagnosis of hypertrophic pylorus stenosis, although it is a well-known fact that ultrasound itself is still not available in many parts of the world [36,37]. In addition, in many parts of the less-developed world, there is a lack of qualified personnel who know how to work with ultrasound, and even when physicians who know how to work with ultrasound are found, the question arises as to how skilled they are in diagnosing hypertrophic pyloric stenosis in newborns. Regarding the ultrasound itself, Al-Jazaeri et al. [24] asked whether metabolic disorders in hypertrophic stenosis of the pylorus are better correlated with the severity of the stenosis, i.e., the thickness and length of the muscle itself. To date, we have not found a study that would answer this question, and our results have shown that the correlation is weak or non-existent.

### Limitations of Study

The study has limitations primarily in the size of the patient sample. The reason for the small sample comes from the fact of the low incidence of hypertrophic pyloric stenosis and the fact that the study included one tertiary center. In addition, the study is retrospective, which means that certain useful indicators, which were not adequately recorded, were lost (e.g., whether there was biliary content in the vomit, a clinical sign of the presence of an olive mass). Furthermore, an ultrasound was performed by several radiologists during the observed period. The results would certainly be more valid if the ultrasound was performed by a single radiologist.

## 5. Conclusions

Given that we have determined that there is a statistically significant difference in certain acid-base variables, such as pH, bicarbonate, total carbon dioxide, base excess, potassium, ionized calcium, and glucose, and in the context of variables such as family history, duration of symptoms, weight at an examination, the difference in weight between birth and examination, and type of vomiting, there is no doubt that the diagnostic potential of acid-base status exists, especially considering the fact that in many parts of the world ultrasound and radiologists are still not widely available. Additional prospective multicenter studies and meta-analyses will certainly be needed to confirm our conclusion more firmly in the future.

## Figures and Tables

**Table 1:** Differences between Q40.0 and R11.0 groups.

Variable	Pylorostenosis Group (Q40.0)	Control Group (R11.0)	*p*-Value
Sex (male, female)	41, 12	41, 12	1.000
Age (days)	31 (24–44)	35 (21–50)	0.674
Duration of symptoms (days)	4 (2–7)	2 (1–5)	0.002 *
Family history	1	0	<0.0001 *
Birth (preterm, term, post-term)	5, 48, 0	6, 47, 0	0.563
Birth weight (grams)	3506 (±558)	3428 (±525)	0.464
Weight during examination (grams)	3880 (±601)	4439 (±1025)	0.001 *
Difference between birth weight and weight during examination (grams)	374 (±568)	1010 (±858)	<0.0001 *
Type of vomiting (explosive, regurgitation)	45, 8	22, 31	0.023 *

* *p* < 0.05.

**Table 2:** Comparison of acid-base status between groups.

Variable	Pylorostenosis Group (Q40.0)	Control Group (R11.0)	*p*-Value	Reference Interval
pH	7.457 (7.425–7.517)	7.422 (7.403–7.446)	<0.0001 *	7.350–7.450
pCO_2_ (kPa)	4.49 (4.16–5.10)	4.70 (4.22–5.10)	0.604	3.60–5.50
pO_2_ (kPa)	8 (7.2–8.7)	8.1 (7.1–8.8)	0.867	4.8–10.6
HCO_3_ (mmol/L)	25 (23–27)	23 (22–24)	0.000 *	19–24
Total CO_2_ (mmol/L)	25 (23–28)	24 (22–25)	0.011 *	22–29
BE (mmol/L)	0.8 (−1.2–2.9)	−1.3 (−2.7–−0.2)	0.000 *	−7–−1
sO_2_ (%)	95.1 (93.3–96.0)	94.1 (91.3–95.7)	0.162	94–98
Potassium (mmol/L)	5 (4.2–5.6)	5.3 (5–5.9)	0.006 *	3–7
Sodium (mmol/L)	137 (136–138)	137 (136–138)	0.278	134–142
Chloride (mmol/L)	103 (100–105)	104 (103–106)	0.071	96–111
Ionized Calcium (mmol/L)	1.28 (1.25–1.31)	1.31 (1.27–1.34)	0.011 *	0.95–1.50
Glucose (mmol/L)	4.5 (4.1–4.8)	4.9 (4.5–5.5)	0.007 *	2.8–5.5
Lactate (mmol/L)	1.98 (1.39–2.57)	1.75 (1.52–2.21)	0.509	0–1.8
Total bilirubin (µmol/L)	<34 (<34–87)	<34 (<34–54)	0.143	<34
Oxyhemoglobin (%)	93.1 (91.5–94.4)	91.9 (89.9–94.3)	0.138	94–100
Carboxyhemoglobin (%)	0.7 (0.5–1)	0.8 (0.4–1)	0.682	<1.5
Methemoglobin (%)	1 (0.9–1.1)	1.1 (0.9–1.2)	0.132	<1.5
Deoxyhemoglobin (%)	4.8 (4–6.6)	5.9 (4.2–8.5)	0.159	<5

* *p* < 0.05, pH—potential of hydrogen, pCO_2_—partial pressure of carbon dioxide, pO_2_—partial pressure of oxygen, HCO_3_—bicarbonate, CO_2_—carbon dioxide, BE—base excess, sO_2_—oxygen saturation.

**Table 3:** Influence of the duration of vomiting on acid-base status in the pylorostenosis group.

Variable	<3 Days (*n* = 23)	>3 Days (*n* = 30)	*p*-Value
pH	7.456 (±0.059)	7.481 (±0.059)	0.123
pCO_2_ (kPa)	4.502 (±0.658)	4.630 (±0.770)	0.525
HCO_3_ (mmol/L)	24.261 (±1.839)	26.700 (±4.419)	0.016 *
Total CO_2_ (mmol/L)	24.174 (±2.249)	26.533 (±5.204)	0.048 *
BE (mmol/L)	−0.161 (±2.174)	2.307 (±4.923)	0.030 *
Potassium (mmol/L)	5.234 (±0.671)	4.633 (±0.987)	0.015 *
Sodium (mmol/L)	137.913 (±2.466)	137.400 (±2.027)	0.410
Chloride (mmol/L)	103.565 (±4.620)	101.633 (±6.636)	0.239

* *p* < 0.05, pH—potential of hydrogen, pCO_2_—partial pressure of carbon dioxide, HCO_3_—bicarbonate, BE—base excess.

**Table 4:** Influence of the duration of vomiting on acid-base status in the control group.

Variable	<3 Days (*n* = 38)	>3 Days (*n* = 15)	*p*-Value
pH	7.424 (±0.036)	7.439 (±0.047)	0.227
pCO_2_ (kPa)	4.601 (±0.637)	4.826 (±0.519)	0.229
HCO_3_ (mmol/L)	23.105 (±1.737)	24.733 (±3.432)	0.026 *
Total CO_2_ (mmol/L)	23.132 (±2.580)	25.133 (±3.889)	0.033 *
BE (mmol/L)	−1.668 (±2.124)	0.267 (±3.694)	0.020 *
Potassium (mmol/L)	5.374 (±0.588)	5.487 (±0.689)	0.551
Sodium (mmol/L)	136.947 (±1.610)	137.200 (±1.568)	0.606
Chloride (mmol/L)	104.184 (±2.276)	103.400 (±5.422)	0.458

* *p* < 0.05, pH—potential of hydrogen, pCO_2_—partial pressure of carbon dioxide, HCO_3_—bicarbonate, BE—base excess.

## Data Availability

The data that support the findings of this study are available upon request from the corresponding author.
